# Supplementary material for: Comparative genomics provides new insights into the diversity, physiology, and sexuality of the only industrially exploited tremellomycete: Phaffia rhodozyma
Source: BMC Genomics. 2016 Nov 9;17:901. doi: 10.1186/s12864-016-3244-7 (PMC5103461; doi:10.1186/s12864-016-3244-7)
Supplement: Additional file 6: — List of orphan genes with links to PFAM (related to Additional file 1: Table S1). (ZIP 1428 kb) [file 12864_2016_3244_MOESM6_ESM.zip › BLAST_HTML_FTR/G04543_P.html]

BLAST Search Results


```
BLASTP 2.2.27+


Reference:
Stephen F. Altschul, Thomas L. Madden, Alejandro A. Schäffer,
Jinghui Zhang, Zheng Zhang, Webb Miller, and David J. Lipman (1997),
"Gapped BLAST and PSI-BLAST: a new generation of protein database
search programs", Nucleic Acids Res. 25:3389-3402.


Reference for
composition-based statistics:
Alejandro A. Schäffer, L. Aravind, Thomas L. Madden, Sergei
Shavirin, John L. Spouge, Yuri I. Wolf, Eugene V. Koonin, and
Stephen F. Altschul (2001), "Improving the accuracy of PSI-BLAST
protein database searches with composition-based statistics and
other refinements", Nucleic Acids Res. 29:2994-3005.


Database: nr
           71,551,133 sequences; 26,053,659,533 total letters


Query= G04543_P

Length=286
                                                                      Score     E
Sequences producing significant alignments:                          (Bits)  Value

emb|CDZ97515.1|  hypothetical protein [Xanthophyllomyces dendrorh...   582    0.0  
emb|CUH91667.1|  putative membrane protein [Herbinix sp. SD1D]        40.0    2.1  
gb|EMG50463.1|  hypothetical protein G210_4105 [Candida maltosa X...  39.3    3.5  


 >emb|CDZ97515.1| hypothetical protein [Xanthophyllomyces dendrorhous]
Length=285

 Score =  582 bits (1500),  Expect = 0.0, Method: Compositional matrix adjust.
 Identities = 285/285 (100%), Positives = 285/285 (100%), Gaps = 0/285 (0%)

Query  1    MSLSFKQINILRAARLIRSSGSGPSAYPSACFVCLKRSQSSSPFSKALIVELEPKRTSVP  60
            MSLSFKQINILRAARLIRSSGSGPSAYPSACFVCLKRSQSSSPFSKALIVELEPKRTSVP
Sbjct  1    MSLSFKQINILRAARLIRSSGSGPSAYPSACFVCLKRSQSSSPFSKALIVELEPKRTSVP  60

Query  61   NSKDGSISDESSSGLSSPPPVPPSKPPPPRISHYSTTSTVSTLCKSLIVSSKDARAGAGF  120
            NSKDGSISDESSSGLSSPPPVPPSKPPPPRISHYSTTSTVSTLCKSLIVSSKDARAGAGF
Sbjct  61   NSKDGSISDESSSGLSSPPPVPPSKPPPPRISHYSTTSTVSTLCKSLIVSSKDARAGAGF  120

Query  121  LTGFVKNPYPLELKYEEYPDGGPSPQEWALIQEFMQEQDEERSLHANPPAILIDSPKSSD  180
            LTGFVKNPYPLELKYEEYPDGGPSPQEWALIQEFMQEQDEERSLHANPPAILIDSPKSSD
Sbjct  121  LTGFVKNPYPLELKYEEYPDGGPSPQEWALIQEFMQEQDEERSLHANPPAILIDSPKSSD  180

Query  181  PHSRNSKLRTFTLPTIVNWTTRQVFYGPYIYSQVEQMIKDMQIEQTERFAAEAKYDAYLR  240
            PHSRNSKLRTFTLPTIVNWTTRQVFYGPYIYSQVEQMIKDMQIEQTERFAAEAKYDAYLR
Sbjct  181  PHSRNSKLRTFTLPTIVNWTTRQVFYGPYIYSQVEQMIKDMQIEQTERFAAEAKYDAYLR  240

Query  241  SQGRRPPSRKKPETDRIKWWEVGLLAIGSYVALDWFYQVWMVKDE  285
            SQGRRPPSRKKPETDRIKWWEVGLLAIGSYVALDWFYQVWMVKDE
Sbjct  241  SQGRRPPSRKKPETDRIKWWEVGLLAIGSYVALDWFYQVWMVKDE  285


>emb|CUH91667.1| putative membrane protein [Herbinix sp. SD1D]
Length=451

 Score = 40.0 bits (92),  Expect = 2.1, Method: Compositional matrix adjust.
 Identities = 33/118 (28%), Positives = 50/118 (42%), Gaps = 18/118 (15%)

Query  168  PPAILIDSPKSSDPHSRNSKLRTFTLPTIVNWTTRQVFY-----------GPYIYSQVEQ  216
            P  IL D  +    H  N   +  TL T  N    + FY           G YIY +  +
Sbjct  128  PGYILEDKERILKAHIDNFAGQDKTLLTYDNMEKEEAFYFYENGALCRQEGYYIYYEKNE  187

Query  217  MIKDMQIEQTERFAAEAKYDAYLRSQGRRPPSRKKPETD-------RIKWWEVGLLAI  267
             ++D  IE  ++ + EA+YD  +  + R     KKP+TD       R+ +    LLA+
Sbjct  188  KMQDYMIEHKQKPSEEAQYDDRVSREIRTIFENKKPQTDDENKSINRLMYAAGTLLAV  245


>gb|EMG50463.1| hypothetical protein G210_4105 [Candida maltosa Xu316]
Length=370

 Score = 39.3 bits (90),  Expect = 3.5, Method: Compositional matrix adjust.
 Identities = 29/96 (30%), Positives = 45/96 (47%), Gaps = 2/96 (2%)

Query  142  GPSPQEWALIQEFMQEQDEERSLHANPPAILIDSPKSSDPHSRNSKLRTFTLPTIVNWTT  201
            GP      ++Q  M E      L A+    ++ S ++ DP  R+  + T   P + NW +
Sbjct  256  GPVRGSATVLQGRMIEHIAPVPLGASERITMVTSYRAKDPKIRDGSVLTTIKPEL-NWGS  314

Query  202  R-QVFYGPYIYSQVEQMIKDMQIEQTERFAAEAKYD  236
            R   FY  +I  +VE M K + I   E+F A  K+D
Sbjct  315  RYNQFYSEWIDYRVELMKKKLDIIVREKFDASGKFD  350


Lambda      K        H        a         alpha
   0.316    0.131    0.398    0.792     4.96 

Gapped
Lambda      K        H        a         alpha    sigma
   0.267   0.0410    0.140     1.90     42.6     43.6 

Effective search space used: 2108778078092


  Database: nr
    Posted date:  Sep 23, 2015 12:05 AM
  Number of letters in database: 26,053,659,533
  Number of sequences in database:  71,551,133


Matrix: BLOSUM62
Gap Penalties: Existence: 11, Extension: 1
Neighboring words threshold: 11
Window for multiple hits: 40
```
